# Postacute Care for Medicare Advantage Enrollees Who Switched to Traditional Medicare Compared With Those Who Remained in Medicare Advantage

**DOI:** 10.1001/jamahealthforum.2023.5325

**Published:** 2024-02-16

**Authors:** Peter J. Huckfeldt, Victoria Shier, José J. Escarce, Brendan Rabideau, Tyler Boese, Helen M. Parsons, Neeraj Sood

**Affiliations:** 1 Division of Health Policy and Management, University of Minnesota School of Public Health, Minneapolis; 2 Leonard D. Schaeffer Center for Health Policy & Economics, University of Southern California, Los Angeles; 3 Division of General Internal Medicine and Health Services Research, Department of Medicine, David Geffen School of Medicine, University of California, Los Angeles; 4 Department of Health Policy and Management, Fielding School of Public Health, University of California, Los Angeles; 5 Department of Health Policy and Management, Bloomberg School of Public Health, Johns Hopkins University, Baltimore, Maryland; 6 Analysis Group, Inc, Los Angeles, California; 7 Department of Health Policy and Management, Sol Price School of Public Policy, University of Southern California, Los Angeles

## Abstract

**Importance:**

Medicare Advantage (MA) plans receive capitated per enrollee payments that create financial incentives to provide care more efficiently than traditional Medicare (TM); however, incentives could be associated with MA plans reducing use of beneficial services. Postacute care can improve functional status, but it is costly, and thus may be provided differently to Medicare beneficiaries by MA plans compared with TM.

**Objective:**

To estimate the association of MA compared with TM enrollment with postacute care use and postdischarge outcomes.

**Design, Setting, and Participants:**

This was a cohort study using Medicare data on 4613 hospitalizations among retired Ohio state employees and 2 comparison groups in 2015 and 2016. The study investigated the association of a policy change with use of postacute care and outcomes. The policy changed state retiree health benefits in Ohio from a mandatory MA plan to subsidies for either supplemental TM coverage or an MA plan. After policy implementation, approximately 75% of retired Ohio state employees switched to TM. Hospitalizations for 3 high-volume conditions that usually require postacute rehabilitation were assessed. Data from the Medicare Provider Analysis and Review files were used to identify all hospitalizations in short-term acute care hospitals. Difference-in-difference regressions were used to estimate changes for retired Ohio state employees compared with other 2015 MA enrollees in Ohio and with Kentucky public retirees who were continuously offered a mandatory MA plan. Data analyses were performed from September 1, 2019, to November 30, 2023.

**Exposures:**

Enrollment in Ohio state retiree health benefits in 2015, after which most members shifted to TM.

**Main Outcomes and Measures:**

Received care in an inpatient rehabilitation facility, skilled nursing facility, or home health, or any postacute care; the occurrence of any hospital readmission; the number of days in the community during the 30 days after hospital discharge; and mortality.

**Results:**

The study sample included 2373 hospitalizations for Ohio public retirees, 1651 hospitalizations for other Humana MA enrollees in Ohio, and 589 hospitalizations for public retirees in Kentucky. After the 2016 policy implementation, the percentage of hospitalizations covered by MA decreased by 70.1 (95% CI, −74.2 to −65.9) percentage points (pp), inpatient rehabilitation facility admissions increased by 9.7 (95% CI, 4.7 to 14.7) pp, use of only home health or skilled nursing facility care fell by 8.6 (95% CI, −14.6 to −2.6) pp, and days in the community fell by 1.6 (95% CI, −2.9 to −0.3) days for Ohio public retirees compared with other Humana MA enrollees in Ohio. There was no change in 30-day mortality or hospital readmissions; similar results were found by comparisons using Kentucky public retirees as a control group.

**Conclusions and Relevance:**

The findings of this cohort study indicate that after a change in retiree health benefits, most Ohio public retirees shifted from MA to TM and received more intensive postacute care with no significant change in measured short-term postdischarge outcomes. Future work should consider additional measures of postacute functional status over a longer follow-up period.

## Key Points

**Question:**

What is the association of Medicare Advantage (MA) enrollment with postacute care use and patient outcomes among retired state employees?

**Findings:**

This cohort study using Medicare data including 4613 hospitalizations of retired Ohio state employees found that after a mandatory MA plan was discontinued, enrollees who switched to traditional Medicare received more intensive postacute care. No changes in 30-day hospital readmissions or mortality were observed.

**Meaning:**

This finding suggests that MA plans provided less intensive postacute care than traditional Medicare, with no significant difference in measured short-term outcomes; measures of postacute functional status over a longer follow-up period are needed.

## Teaser

This cohort study estimates the association of Medicare Advantage compared with traditional Medicare enrollment with postacute care use and postdischarge outcomes among state retirees in Ohio.

## Introduction

The share of Medicare beneficiaries enrolled in Medicare Advantage (MA) instead of traditional fee-for-service Medicare (TM) continues to grow, with the MA share increasing from 19% in 2007 to 51% in 2023.^1^ The US Centers for Medicare & Medicaid Services (CMS) pay MA plans a monthly capitated rate to cover nearly all health care expenses for plan enrollees, and plans keep as profits the portion of the payment not used for enrollees’ health care expenses. Plans are permitted to use tools such as prior authorization and a limited provider network to manage enrollees’ health care use.^2^ Proponents argue that these financial incentives and delivery tools for MA plans enable them to provide care more efficiently than TM. However, in reducing costs, MA plans may focus on maintaining short-term health outcomes while neglecting services that promote better long-term health and functioning.

Postacute care (PAC) may contribute to better long-term functioning, but also imposes higher short-term costs, and thus may be provided differently by MA plans compared with TM. After a hospital stay, patients can receive rehabilitation services and skilled nursing care in an inpatient rehabilitation facility (IRF), a skilled nursing facility (SNF), or in their home from a home health agency (HH). Although there have not been large randomized clinical trials evaluating alternative settings for postacute rehabilitation services, observational research has found that patients admitted to IRFs after a hospitalization for stroke had better mobility and ability to perform self-care compared with patients admitted to SNFs.^3^ Other research found that patients admitted to IRFs had lower mortality (after stroke hospitalizations) and increased longer-term community residence (after hip fracture and stroke hospitalizations) compared with patients discharged to SNFs.^4^ Similarly, patients admitted to SNFs had lower readmissions than patients receiving HH, although with no difference in mortality or functional outcomes.^5^ However, IRF stays are substantially more expensive than SNF stays, which in turn are more expensive than HH episodes.^5,6^

A 2022 report from the US Department of Health and Human Services Office of the Inspector General found that MA plans had denied requests for IRF care that were deemed to meet criteria for medical necessity.^6^ Consistent with the report, prior cross-sectional research has found that MA enrollees were more likely than TM enrollees to be discharged to home without institutional postacute or HH after hospitalizations for joint replacement, stroke, and heart failure,^7^ and were less likely than TM enrollees to be admitted to IRFs across conditions.^7,8^ The prior cross-sectional studies found limited evidence of an adverse association between MA enrollment and postdischarge outcomes such as readmissions. However, MA enrollment is voluntary and MA enrollees tend to be healthier (ie, have lower mortality risk) than TM enrollees conditional on age, sex, and Medicaid enrollment.^9^ As a result, studies relating observed MA enrollment with patient outcomes may overstate MA’s ability to reduce costs without harming patient health. This study builds on prior work using changes in state retiree health benefits in Ohio as a natural experiment to estimate the association of MA enrollment with PAC use and postdischarge outcomes.

## Methods

This study was reviewed and approved by the University of Minnesota Institutional Review Board. Informed consent was waived because the research involved minimal risk to participants. The study followed the Strengthening the Reporting of Observational Studies in Epidemiology (STROBE) reporting guideline.

We investigated changes in PAC use and postdischarge outcomes among retired Ohio state employees (hereafter, public retirees) whose retiree health benefits changed from a mandatory sponsored MA plan for public retirees in 2015 to a subsidy for purchasing coverage through an MA plan or supplemental coverage for TM in 2016. In a 2015 benefits guide, the Ohio Public Employee Retirement System (OPERS) explained the change by noting that the OPERS-sponsored MA plan was more costly than the more comprehensive supplemental Medigap plans on the individual market and that per-retiree costs of providing the OPERS group plan were rising.^10^ Under the new OPERS retiree health benefit, public retirees received a monthly Health Reimbursement Account allowance of up to $337 that they could use to purchase Medigap, Part D, or MA coverage through a private Medicare exchange (the Medicare Connector) managed by a private contractor. In January 2015, all Medicare-eligible Ohio public retirees receiving health benefits were enrolled in a mandatory group MA plan managed by Humana, Inc (hereafter, other Humana MA enrollees). In 2016, when public retirees had a choice of supplemental TM or MA plans, only 25% chose to remain enrolled in MA (eResults in Supplement 1). The OPERS benefits guide describing the comprehensive benefits of supplemental TM Medigap plans may have persuaded some public retirees to switch to TM.

We estimated intent-to-treat effects of this policy change using a difference-in-differences approach, comparing changes in outcomes for Ohio public retirees after the change in health benefits with comparison groups that were unaffected by the change. We focused on 2 distinct comparison groups that were likely to provide an accurate counterfactual for what would have happened to public retirees in the absence of the changes in health benefits. Comparison group 1 comprised Ohio enrollees in other Humana MA plans (ie, not the plan for Ohio public retirees) in January 2015, 98% of whom remained enrolled in MA in 2016 as well. Because public retirees may have different trends in health care use than other Medicare beneficiaries, for comparison group 2 we chose public retirees in Kentucky because they received retiree health benefits through a mandatory Humana MA plan in 2015 and 2016. Given that 75% of the treatment group in Ohio shifted from MA to TM, whereas no more than 2% of the comparison groups shifted from MA to TM, our intention-to-treat estimates may provide a conservative estimate of the association of MA enrollment with the stated study outcomes.

### Study Population

Our analysis investigated hospital stays and 30-day postdischarge periods for the treatment group and the 2 comparison groups between 2015 and 2016. We focused on 3 high-volume conditions that require intensive rehabilitation after hospitalization: lower extremity joint replacement after a fracture; hip and femur procedures (after a fracture; in an academic tertiary hospital, the most common procedures were intramedullary nailing, dynamic hip screw, and open reduction internal fixation^11^); and stroke (only the first stroke observed per patient during the study period). For joint replacement, we included MS-DRG codes 469 and 470, with a principal diagnosis of fracture per the *International Classification of Diseases, Ninth Revision (ICD-9) and 10 Revision (ICD-10)* that are used by the CMS Comprehensive Joint Replacement program^12^; and for stroke, we included MS-DRG codes 64, 65, and 66. Medicare stipulates that 60% of IRF admissions must meet a set of conditions determined to require intensive rehabilitation; hip fracture and stroke are both included.^13^ Notably, these hospitalizations are unlikely to be elective and are not readily predictable as they occur after an acute event such as a fracture or a stroke. Therefore, it is unlikely that MA compared with TM enrollment will substantially affect the probability of hospitalization for these conditions, although we also investigated this empirically. We excluded hospital discharges occurring in December 2016 because we were not able to observe PAC use in 2017. Our analysis focused on index hospitalizations—admissions that did not occur within 30 days of another hospital discharge—and excluded hospitalizations during which the patient died.

### Data Sources

We used the Medicare Master Beneficiary Summary File to identify retirees in Ohio with an MA plan or TM coverage, MA plan type and contract identifiers (for MA enrollees), state of residence, demographic and socioeconomic status information, and mortality. We identified the contract identifiers for public retirees using information from the Ohio Public Retirees Open Enrollment Guide for retired state employees, detailed in the eAppendix in Supplement 1.^10,14^

A challenge to comparing health care use between MA and TM enrollees is finding consistent data for both types of Medicare beneficiaries. We identified index hospitalizations using the Medicare Provider Analysis and Review (MedPAR) files, focusing on hospitals that received disproportionate share or medical education payments from CMS that are required to submit encounter claims for MA enrollees to receive full payment^15,16^; notably, this includes most short-term acute care hospitals.^7^ We identified hospital discharges for the included conditions based on the Medicare Severity Diagnosis-Related Group (MS-DRG) on the hospital claim.^12^

We identified SNF admissions and days using SNF claims for TM enrollees and the Minimum Data Set (MDS) assessments for MA enrollees (augmented with SNF claims, in the small number of cases where they were reported in MedPAR). We identified IRF stays from the Inpatient Rehabilitation Facility Patient Assessment Instrument, and HH episodes from the Home Health Outcome and Assessment Information Set (OASIS) data given that facilities must submit assessments for both MA and TM enrollees.^17^

For our primary approach for measuring hospital readmissions, we used MedPAR and identified all hospitalizations in short-term acute care hospitals (across conditions) occurring within 30 days of the index hospital discharge for which hospitals are required to submit information-only claims for MA enrollees. We created an alternative measure of readmissions, augmenting the MedPAR hospitalization data with discharge-level data from the Healthcare Effectiveness Data and Information Set (HEDIS), which is further described in the eAppendix in Supplement 1.

### Study Measures

The outcome variables for the study analyses included measures of PAC use and patient outcomes within 30-days of hospital discharge. We focused on 3 binary categories indicating level of PAC use: patient received any IRF, with or without SNF and/or HH (category 1); SNF or HH, but no IRF (category 2); and no PAC, ie, no HH, SNF, or IRF (category 3). For category 2, we separately assessed whether the patient had received SNF (with or without HH) compared with receiving only HH. Patient outcome variables included whether patients were readmitted to a hospital for any condition within 30 days of discharge (constructed separately using solely MedPAR data, and using both MedPAR and HEDIS data); the number of days in the community during the 30-day postdischarge period (ie, alive and not in a hospital, nursing home, or institutional PAC setting); the number of days in an SNF, IRF, or nursing home; the number of days in a hospital; and whether death occurred during the 30 days postdischarge; and days deceased.

### Statistical Analyses

Difference-in-differences regressions were used to estimate changes in PAC use and postdischarge outcomes after hospitalizations before and after the benefits policy change compared with each of the 2 comparison groups. Explanatory variables in the regression included the interaction effect of being an Ohio public retiree after the policy change in 2016 (giving the coefficient estimate of interest), an indicator variable for being an Ohio public retiree, year by quarter fixed effects, hospital fixed effects, and control variables, including age, sex, RTI (Research Triangle Institute) race and ethnicity code, MS-DRG code (ie, reason for a hospital admission), Medicaid eligibility, and fixed effects for the discharging hospital. We did not control for other comorbidities because of concerns about more intensive diagnosis coding in MA.^18^ We estimated separate models for each of the 2 comparison groups. Linear regressions were used for continuous variables and linear probability models were used for binary outcomes. Clustered standard errors were calculated at the treatment status by health service area level.^19^ Further details on the statistical analysis are available in the eAppendix in Supplement 1.

In preliminary analyses, we estimated difference-in-differences regressions at the person-quarter level to assess whether the policy shifting Ohio public retirees to TM changed the probability of having hospital admissions for the 3 conditions in our defined cohort. We performed this initial analysis because inclusion in our main analyses required having a hospitalization for a set of conditions, which could introduce selection bias if MA enrollment affects the probability of hospital admission for these conditions.

The eAppendix in Supplement 1 describes event study regressions that allowed for testing for differences in time trends in outcomes by treatment status before the policy implementation and assess dynamic estimated effects on outcomes postpolicy intervention. Intent-to-treat estimates included Ohio public retirees who shifted to TM but also a minority number of individuals who continued to be enrolled in MA. To understand the broader generalizability of these estimates, we compared the characteristics of the 2 groups of Ohio public retirees (those who in 2016 switched from mandatory MA plan to other MA plans vs those who switched to TM instead). As a placebo test, we estimated separate difference-in-difference regressions for Ohio public retirees who continued with the MA plan (vs those who switched to TM). In addition, we explored the presence of heterogeneous effects, estimating separate effects for patients with stroke or fracture, and for patients residing in metropolitan or nonmetropolitan counties.^20^

Statistical tests were 2-tailed and *P* < .05 were considered statistically significant. Data analyses were performed from September 1, 2019, to November 30, 2023, and final estimates were generated using Stata, version 18.0 MP-parallel edition (StataCorp).

## Results

From January 1 to December 31, 2015, and from January 1 to November 1, 2016, there were 1217 and 1156 hospital discharges, respectively, for the 3 conditions (lower extremity joint replacement after a fracture, hip and femur procedures, and stroke) among Ohio public retirees compared with 840 and 811 for other Ohio Humana MA enrollees, and 285 and 304 for Kentucky public retirees (Table 1). The demographic characteristics of the Ohio public retirees in 2015 were similar to those of the comparison groups; eg, both Ohio and Kentucky, public retirees were more likely to be female. However, in Kentucky, a higher percentage of public retirees were White. The composition of hospitalized individuals did not change substantially among groups across the 2 years. Notably, we found no relative change in the probability of hospitalization for the conditions in the study sample for Ohio public retirees and either of the comparison groups (other Ohio Humana MA enrollees or Kentucky retirees), alleviating concerns about selection bias coming from conditioning the main analysis on having a hospitalization (eResults in Supplement 1).

**Table 1.** Comparison of Characteristics of Hospitalized Retirees With Public Insurance, by Study Group, 2015 vs 2016

Characteristic	No. (%)					
	Treatment group		Comparison group 1		Comparison group 2	
	Ohio public retirees, 2015		Ohio other Humana MA enrollees, 2015		Kentucky public retirees, 2015	
	2015 (n = 1217)	2016 (n = 1156)	2015 (n = 840)	2016 (n = 811)	2015 (n = 285)	2016 (n = 304)
Age, mean (SD), y	81.4 (8.1)	81.4 (8.2)	79.4 (8.1)	79.2 (7.6)	79.1 (8.0)	79.6 (7.5)
Female	806 (66.2)	768 (66.4)	523 (62.3)	481 (59.3)	189 (66.3)	205 (67.4)
Male	411 (33.8)	388 (33.6)	317 (37.7)	330 (40.7)	96 (33.7)	99 (32.6)
Race and ethnicity						
Black, non-Hispanic	102 (8.4)	98 (8.5)	72 (8.6)	84 (10.4)	NR	NR
White, non-Hispanic	1096 (90.1)	1035 (89.5)	755 (89.9)	712 (87.8)	267 (93.7)	284 (93.4)
Other^a^	19 (1.6)	23 (2.0)	13 (1.5)	15 (1.8)	NR	NR
Dual eligible	49 (4.0)	35 (3.0)	88 (10.5)	62 (7.6)	NR	NR
Index hospitalization						
Stroke	612 (50.3)	610 (52.8)	453 (53.9)	430 (53.0)	135 (47.4)	148 (48.7)
Fracture (without joint replacement)	404 (33.2)	351 (30.4)	253 (30.1)	259 (31.9)	98 (34.4)	99 (32.6)
Joint replacement due to fracture	201 (16.5)	195 (16.9)	134 (16.0)	122 (15.0)	52 (18.2)	57 (18.8)

Abbreviations: MA, Medicare Advantage; NR, not reported for small sample size.

^a^ Included unknown, American Indian or Alaska Native, Asian, Pacific Islander, Hispanic, and other.

### Descriptive Findings

The adjusted rate of MA coverage for hospital stays (adjusting for the control variables) decreased for Ohio public retirees hospitalized for the 3 conditions, from nearly 100% in early 2015 to approximately 25% in 2016, whereas enrollees in other Ohio Humana MA plans in 2015 (comparison group 1) mostly continued their enrollment in MA in 2016 (Figure, A). When Ohio public retirees’ health benefits were provided through a mandatory Humana MA plan in 2015, inpatient rehabilitation use was similar to other Ohio Humana MA plans (<8% of discharges), but when most Ohio public retirees switched to TM in 2016, inpatient rehabilitation admissions increased to approximately 16% of hospital discharges, whereas other Ohio Humana MA plans’ IRF admission rates remained lower (Figure, B).

**Figure.**
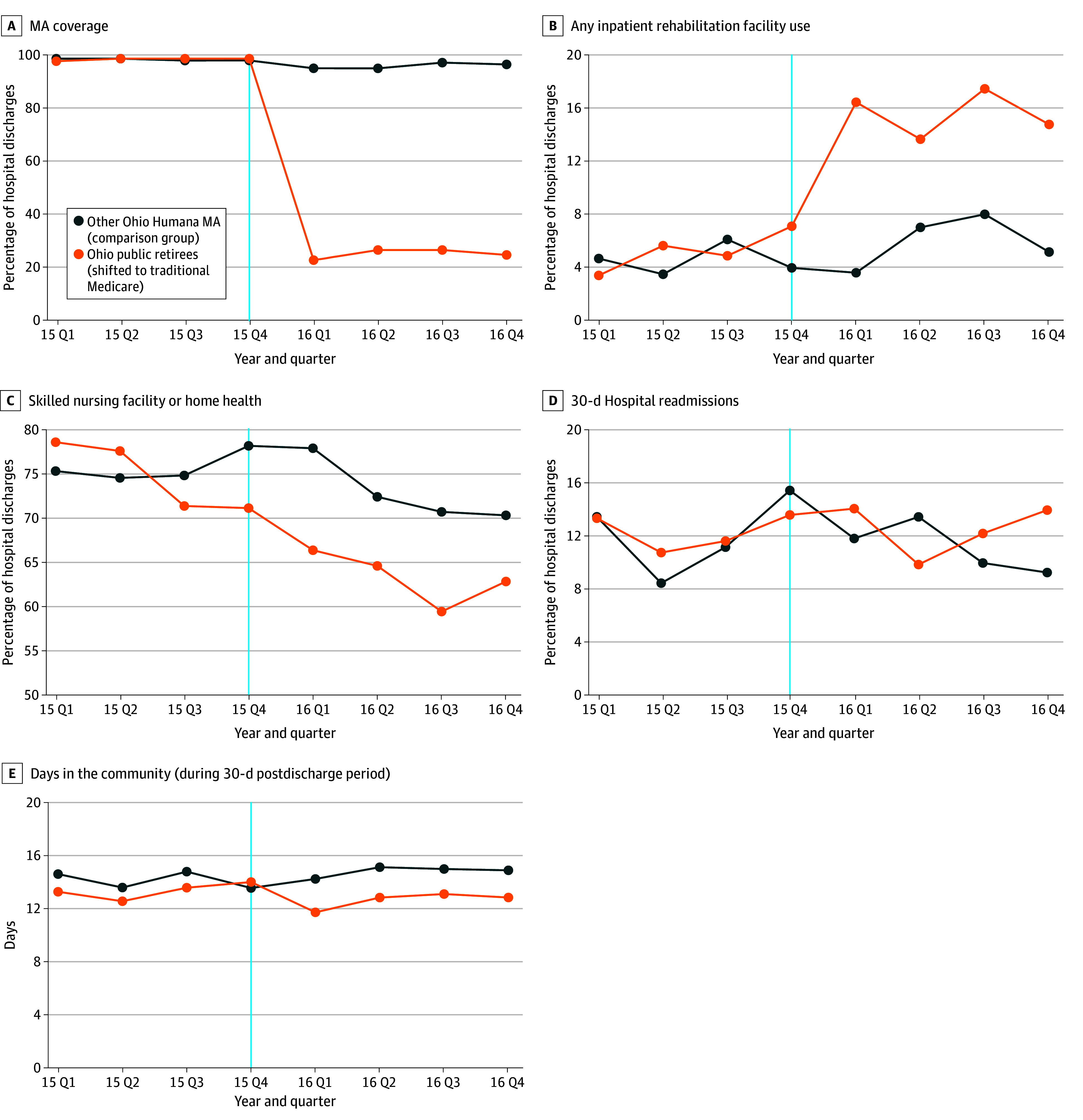
Adjusted Medicare Advantage (MA) Coverage, Postacute Care Use, and Patient Outcomes, 2015-2016 When MA coverage (A) was more than 99% but less than 100%, we plotted 99% to ensure sample sizes greater than 12.

The percentage of Ohio public retirees and other Ohio Humana MA enrollees receiving only HH or SNF was greater than 75% in 2015, but fell for Ohio public retirees in 2016 (Figure, C); the overall high rate of SNF admissions reflects that approximately half of the sample was comprised of patients with hip fracture , a condition that has a high rate of discharge to SNFs after hospitalization.^21^

Hospital readmissions were similar for Ohio public retirees and other Humana MA enrollees in both 2015 and 2016, suggesting that switching from MA to TM did not affect hospital readmissions (Figure, D). However, there was a decrease in the number of days Ohio public retirees spent in the community after hospital discharge after the switch from TM to MA compared with other Ohio MA enrollees (Figure, E). Event study results show that trends in each outcome were generally parallel between Ohio public retirees and other Ohio Humana MA enrollees before the policy change (supporting the use of other Ohio Humana MA enrollees as a comparison group; eResults in Supplement 1). By the fourth quarter of 2016, inpatient rehabilitation facility admissions had increased by 11.5 percentage points (pp) (95% CI, 5.5 to 17.8; *P* < .001) and receiving only HH or SNF fell by 9.8 pp (95% CI, −1.2 to −1.4; *P* = .02).

### Difference-in-Differences Results

Table 2 displays difference-in-differences estimates for PAC and postdischarge patient outcomes. The first column displays the 2015 mean for Ohio public retirees, followed by separate difference-in-differences estimates for Ohio public retirees compared with other Ohio Humana MA enrollees (comparison group 1) and Ohio public retirees compared with Kentucky public retirees (comparison group 2). Compared with other Ohio Humana MA enrollees, the percentage of hospitalizations for Ohio public retirees covered by MA decreased by 70.1 (95% CI, −74.2 to −65.9) pp from 2015 to 2016 (*P* < .001). During the same period, relative to other Ohio Humana MA enrollees, inpatient rehabilitation facility admissions among Ohio public retirees increased by 9.7 (95% CI, 4.7 to 14.7; *P* < .001) pp and the percentage of Ohio public retirees receiving only HH or SNF fell by 8.6 (95% CI, −14.6 to −2.6; *P* = .006) pp; this was nominally driven by reductions in SNF rather than only HH use; however, the estimates were statistically insignificant. There was no change in the overall probability of using PAC. We found similar relative changes in PAC use comparing Ohio public retirees to Kentucky public retirees, although there was a nominally larger, but statistically insignificant, reduction in receiving no PAC, and the reduction in use of SNF or HH without IRF was also statistically insignificant.

**Table 2.** Difference-in-Difference Estimates for Medicare Advantage (MA) Coverage and Postacute Care Use

Outcome	2015 Mean for Ohio public retirees, %	Difference-in-difference estimates for Ohio public retirees vs comparison groups			
		Other Ohio Humana MA (comparison group 1)		Kentucky public retirees (comparison group 2)	
		Estimate (95% CI)	*P* value	Estimate (95% CI)	*P* value
MA coverage	>99	−70.1 (−74.2 to −65.9)	<.001	−73.1 (−77.6 to −68.6)	<.001
Postacute care use					
Any IRF	5.0	9.7 (4.7 to 14.7)	<.001	12.0 (4.3 to 19.6)	.003
SNF or HH (no IRF)	76.2	−8.6 (−14.6 to −2.6)	.006	−7.4 (−17.8 to 3.1)	.17
SNF with or without HH	65.6	−5.8 (−11.9 to 0.3)	.06	−3.8 (−11.8 to 4.1)	.34
Only HH	10.6	−2.8 (−7.2 to 1.6)	.20	−3.5 (−11.3 to 4.2)	.36
No postacute care	18.8	−1.1 (−5.4 to 3.2)	.62	−4.6 (−11.0 to 1.8)	.16
Patient outcomes					
30-d Hospital readmission	12.1	0.9 (−3.1 to 4.9)	.66	2.3 (−2.6 to 7.2)	.36
30-d Hospital readmission (broader, using HEDIS)	13.9	0.4 (−3.5 to 4.3)	.84	1.0 (−6.7 to 6.8)	.98
Death during 30-d postdischarge episode	7.3	1.2 (−1.6 to 4)	.40	−1.4 (−5.8 to 3.1)	.54
Retiree location during 30-d posthospital discharge period, d					
Community	12.8	−1.6 (−2.9 to −0.3)	.02	−2.5 (−4.9 to −0.1)	.04
Institutional postacute care	15.0	1.1 (−0.1 to 2.4)	.08	3.1 (0.9 to 5.3)	.007
Hospital	0.7	0 (−0.1 to 0.2)	.62	−0.2 (−0.5 to 0.2)	.32
Deceased	1.5	0.4 (−0.2 to 1)	.16	−0.4 (−1.4 to 0.6)	.45

Abbreviations: HEDIS, Healthcare Effectiveness Data and Information Set; HH, home health agency; IRF, inpatient rehabilitation facility; SNF, skilled nursing facility.

We assessed 2 measures of readmissions: the first included readmissions identified in MedPAR occurring in short-term acute care hospitals that are required to submit information-only claims to Medicare for MA enrollees. The second included all acute care and critical access hospital readmissions identified in MedPAR as well as hospital readmissions identified in HEDIS data (for MA enrollees). Despite the increased intensity of PAC use after Ohio public retirees switched from MA to TM, we found no relative change in hospital readmissions across readmission measures and comparison groups.

In the 30 days after hospital discharge, the Medicare beneficiaries in the sample were in 1 of 4 mutually exclusive and exhaustive states: (1) in the community, (2) readmitted to hospital, (3) in a PAC facility or nursing home, or (4) deceased. We investigated the number of days-stay in each state for Ohio public retirees and the 2 comparison groups. We found statistically significant reductions in the number of days that Ohio public retirees resided in the community during the first 30 days after hospital discharge (−1.6 [95% CI, −2.9 to −0.3] and −2.5 [95% CI, −4.9 to −0.1]) days for comparison groups 1 and 2, respectively. The reduction in community days was nominally driven by increased days in PAC, but this was only significant when comparing Ohio public retirees with Kentucky public retirees (3.1; 95% CI, 0.9 to 5.3; *P* = .007) days. We also found no significant change in mortality after Ohio public retirees switched to TM.

Our analysis used an intent-to-treat design, and not all Ohio public retirees switched to TM in 2016 (approximately 25% of hospitalized patients in our sample remained in MA). These 2 groups were similar in terms of demographic and clinical composition, implying the broader generalizability of the estimates across Ohio public retirees (eResults in Supplement 1). Changes in PAC use were driven entirely by the Ohio public retirees switching to TM (eResults in Supplement 1), ruling out that effects were driven by changes in plan design among public retirees electing to continue coverage with MA. We found nominally larger estimated effects on PAC use for patients with stroke rather than fractures patients, but the differences in the effects were statistically insignificant (eResults in Supplement 1). We found a similar pattern of results for metropolitan compared with nonmetropolitan counties, although there was nominal (but statistically insignificant) increase in receiving no PAC for nonmetropolitan counties (eResults in Supplement 1).

## Discussion

When Ohio shifted public retiree health benefits from a mandatory MA plan in 2015 to subsidies for either a MA plan or supplemental coverage for TM, most retirees switched to TM. This natural experiment provided a unique opportunity to identify the association of MA with PAC use and outcomes for Medicare beneficiaries. We found that, under TM, Ohio public retirees were more likely to be discharged to a more intensive and expensive IRF and less likely to receive only SNF or HH. However, despite receiving more intensive PAC under TM, hospital readmissions and 30-day mortality for Ohio public retirees were unchanged. Because of the direct mechanical association with more days spent in PAC facilities, days in the community during the 30 days postdischarge were reduced under TM.

The finding that Ohio public retirees were more likely to be discharged to an IRF under TM is consistent with the findings of prior cross-sectional studies comparing MA with TM, which found lower use of PAC overall in MA,^7,8,22^ shorter stays in SNFs with fewer therapy minutes,^23^ lower use of postacute HH,^7,24^ lower use of IRF,^7,8^ and evidence of substitution of SNF for IRF care after stroke and joint replacement hospitalizations.^7,8^

A key contribution of our research is showing that MA vs TM differences in IRF use reflect different approaches to providing PAC rather than patient-level selection into MA. However, an important unanswered question raised by this and previous studies is whether the reduced use of IRFs in MA results from case-by-case “active management” that tries to match each patient to the most appropriate type of PAC or from general restrictions—eg, through the exclusion of IRFs from plans’ networks—that can only be overcome with extensive effort, persistence, and clinical justification. Notably, the available benefits documentation for the sponsored MA plan in our study did not mention IRF services.

Barnett et al^25^ previously noted that TM payment reforms, such as Accountable Care Organizations and bundled payment and MA plans generate savings by reducing the use of PAC, which may have adverse effects on patients and caregivers. Moreover, the concern with the lower use of IRF in MA is that beneficiaries who would benefit from the more intensive rehabilitation services may not be allowed access to these services. We found that when compared with MA, TM did not affect hospital readmissions or 30-day mortality for Ohio public retirees. However, long-term improvement in functioning, the main goal of rehabilitation, may occur even without changes in short-term outcomes such as hospital readmission and 30-day mortality.

As noted earlier, prior studies of TM beneficiaries^3,4^ found that patients with hip fracture or stroke were more likely to be alive and residing in the community at 120 days after hospital discharge when they received rehabilitation care in IRFs compared with SNFs, and that patients admitted to IRFs had better mobility and ability to perform self-care activities than patients in SNFs. Other recent research using the US National Health and Aging Trends Study^22^ found that MA enrollees had less functional improvement during postacute care use. More causal evidence is needed on the effects of MA compared with those of TM for a broader set of functional outcomes over a longer study period.

### Limitations

This analysis had limitations. First, hospital readmissions and community residence at 30 days after discharge do not fully capture postdischarge functional status. Future work should consider the longer-term effects of less-intensive PAC on beneficiaries’ functional status and on the well-being of caregivers. Second, a key unanswered question for future research is the mechanisms by which MA plans change PAC use, eg, whether this is done using prior authorization, more active discharge planning, or as previously suggested, narrower facility and clinician networks that largely exclude IRFs. Third, we selected conditions for which intensive rehabilitation is less discretionary after a hospitalization; the association of MA with PAC use may differ for other conditions requiring less rehabilitation.

## Conclusions

This cohort study found that after a change in retiree health benefit policy, most Ohio public retirees shifted from MA to TM and received more intensive PAC with no significant change in the measured short-term postdischarge outcomes. Future work should consider measures of postacute functional status over a longer follow-up period.

## Notes

Supplement 1. eAppendix eResults.

Supplement 2. Data Sharing Statement
