# Supplementary material for: Postacute Care for Medicare Advantage Enrollees Who Switched to Traditional Medicare Compared With Those Who Remained in Medicare Advantage
Source: JAMA Health Forum. 2024 Feb 16;5(2):e235325. doi: 10.1001/jamahealthforum.2023.5325 (PMC10873769; doi:10.1001/jamahealthforum.2023.5325)
Supplement: Supplement 1. — eAppendix eResults. [file jamahealthforum-e235325-s001.pdf]

Supplemental Online Content

Huckfeldt PJ, Shier V, Escarce JJ, et al. Postacute Care for Medicare Advantage Enrollees Who Switched to Traditional Medicare Compared With Those Who Remained in Medicare Advantage. *JAMA Health Forum*. Published online February 16, 2024. doi:10.1001/jamahealthforum.2023.5325

eAppendix

eResults

This supplemental material has been provided by the authors to give readers additional information about their work.

© 2024 Huckfeldt PJ et al. *JAMA Health Forum*

eAppendix

1. **Identifying Ohio public retirees (Treatment group), Other Ohio Humana enrollees (Comparison Group 1), and Kentucky public retirees (Comparison Group 2)**

While there is extensive publicly available documentation on public retiree MA plan benefits and enrollment, this documentation does not include information on contract and plan IDs to identify plan members in Medicare data. This section outlines how we identified contract and plan IDs for the public retiree MA plans in our study.

Identifying Ohio public retirees

Reviewing publicly available plan documentation for retired Ohio state employees from the Ohio Public Employees Retirement System[i], we were able to ascertain that public retirees were offered a Humana Employer Group PPO MA plan in 2015 (and numerous years prior) and an Express Scripts Medicare Part D plan ((i.e., there was not a Part D plan as a part of the Humana Employer Group MA plan). Starting in 2016, the MA plan and Part D plans were no longer offered. Instead, retirees were offered a subsidy and could choose a Medigap, Medicare Advantage, and/or Part D plan on the "OneExchange OPERS Medicare Connector". [ii]

From the 2015 OPERS Comprehensive Annual Financial Report[iii], we could tell that there were 174,750 retirees and primary beneficiaries enrolled in OPERS health care plans in 2015. Notably, this total includes retirees that were Medicare-eligible and those that are not yet Medicare-eligible (and hence would not be present in our data). While the OPERS plan documentation does not indicate a specific contract and plan ID for the Mandatory MA Plan, CMS MA directory data only listed one Humana MA Contract (H6609) with Employer Group

[i] 2015 Coverage Guide for Participants in the OPERS Health Care Plan.
[ii] OPERS Medicare Connector: Facts every employer should know. June 2015
[iii] OPERS 2015 Comprehensive Annual Financial Report.

© 2024 Huckfeldt PJ et al. *JAMA Health Forum*

Health Plans with enrollment in Ohio in 2015 (H6609) and there was only one plan ID (802) that met the plan's specifications in CMS data (PPO without a Part D plan) and that had comparable enrollment to the annual financial report (accounting for the non-Medicare eligible population).[iv] In addition, we found that over 96% of H6609-802 plan enrollees were also enrolled in an Express Scripts Part D plan. Moreover, we found that this plan had a drastic reduction in enrollment in 2016 and 2017 (consistent with the plan remaining to be available to small number of Medicare-eligible re-employed retirees, retirees enrolled in Medicare Part B, but not yet eligible for premium-free Medicare Part A, and Medicare-eligible retirees under age 65 with end-stage renal disease after it was discontinued for most employees).[v]

Identifying other Ohio Humana MA enrollees

We included Humana contracts with at least 11 Ohio enrollees in January 2015. These include the following contract IDs: H1019 H1036 H2012 H2649 H4461 H4510 H5525 H5415 H8145 H8953 R5826. We excluded one Humana contract that was for Private Fee-for-service coverage (H8145) and another that appeared to be discontinued in 2016 (H5415). We also included enrollees in other plans (excluding the Ohio public retiree plan) within contract H6609, except we excluded one plan (802) that lost nearly all of its enrollment in 2016.

Identifying Kentucky public retirees (comparison group)

The Kentucky Retirement System (KRS) provided coverage through a mandatory Humana Medicare Advantage plan starting January 1, 2013.[vi] The 2015 KRS Comprehensive Annual

---

[iv] January 2015 – Monthly Enrollment by Contract/Plan/State/County. Available at: https://www.cms.gov/Research-Statistics-Data-and-Systems/Statistics-Trends-and-Reports/MCRAdvPartDEnrolData/Monthly-Enrollment-by-Contract-Plan-State-County

[v] The OPERS Medicare Connector (2016).

[vi] Kentucky Retirement System. Public Pension Oversight Board. Retiree Healthcare. September 26, 2016. https://www.kyret.ky.gov/About/Board-of-Trustees/PublicPensionOversightBoardMaterials/September262016KRSHealthcarePresentation.pdf

© 2024 Huckfeldt PJ et al. *JAMA Health Forum*

Report lists that there were 4,019 Medicare enrollees without Part D coverage and 44,748 with prescription drug coverage as of June 30, 2015.[vii] There were only two major Humana MA Contracts with Employer Group Health Plans with enrollment in Kentucky greater than 1000 enrollees in 2015. One had only ~8500 enrollees and the other had 41,339 enrollees which we ascertained to be the Kentucky public retiree plan. We focused on H6609-801 which included a Part D plan to make the comparison group more comparable to Ohio public retirees, who were offered a Part D plan.

2. Alternative hospital readmission measure

There is some concern that MedPAR data may not capture hospitalizations for all hospitals and that there is less of a financial incentive for hospitals to report MA encounter claims relative to TM admissions. MA plans are required to report HEDIS measures on hospital admissions and readmissions to the National Committee on Quality Assurance for quality reporting purposes. These data do not provide information on the reason for a hospitalization, and as a result we could not identify additional "index" hospital discharges for the conditions in our sample, but the HEDIS data do include admission dates that allowed us to capture additional hospital readmissions. Using these data, we constructed a second readmission measure that identified all hospital readmissions in MedPAR data, including those occurring critical access hospitals not required to submit information-only claims for MA enrollees, as well as readmissions identified in HEDIS data. The HEDIS data exclude some readmissions, but including readmissions reported in either MedPAR or HEDIS might more accurately estimate the census of readmissions.[1] We performed our main analysis on the CMS Virtual Research Data Center, but the analysis of the readmission measure including HEDIS data was estimated using physical data

---

[vii] Kentucky Retirement System. Comprehensive Annual Financial Report 2015

© 2024 Huckfeldt PJ et al. *JAMA Health Forum*

on a server at the University of Southern California (USC). The underlying MedPAR data were slightly different on the VRDC versus the USC server leading the samples to differ slightly (2373 versus 2382 index admissions for Ohio public retirees, 1651 versus 1657 admissions for other Ohio Humana enrollees, and 591 versus 589 admissions for Kentucky public retirees) but estimates for all other outcomes were very similar between the two samples.

3. Differences-in-differences and event study models

We estimated differences-in-differences regressions modeling each study outcome as a function of whether hospitalized beneficiaries were Ohio public retirees in the post-policy change period (2016) and controlled for age, sex, race and ethnicity, the Medicare Severity-Diagnosis Related Group (i.e., the reason for a hospital admission), Medicaid eligibility, and hospital fixed effects. We did not control for other comorbidities because of concerns about more intensive diagnosis coding in MA.

Equation 1 displays our main regression specification:

$$y_{ight} = \alpha + \beta_h + \gamma_t + X'_{it}\eta + \lambda Treat_g + \theta Treat_g \times POST_t + u_{ight} \quad (1)$$

Y is an outcome for patient i from treatment or comparison group g, from hospital h, in quarter t; α is a constant term, β are hospital fixed effects, γ are quarter-year fixed effects, *Treat* is an indicator for being a 2015 Ohio public retiree, *X* are control variables, and θ is the estimate of interest, the differential change in an outcome for Ohio public retirees compared to each comparison group. We estimated separate models for each of the two comparison groups.

We also estimated event study models which are similar to Equation 1 but interact "Treat" with each year by quarter time indicator (instead of POST) The event study used the quarter before the policy change as the reference period and estimated differences in outcomes for Ohio public retirees and Comparison Group 1 in each quarter relative to the difference in

© 2024 Huckfeldt PJ et al. *JAMA Health Forum*

outcomes for Ohio public retirees and Comparison Group 1 in the reference quarter. This specification allowed us to test for differences in time trends in outcomes prior to policy implementation and also we examined dynamic effects on outcomes post policy intervention.

We calculated clustered standard errors at the level of treatment, which in this case is treatment status (i.e., Ohio public retiree versus Comparison Group 1 or 2) by Health Service Area (defined by the National Center for Health Statistics to be "a single county or cluster of contiguous counties which are relatively self-contained with respect to hospital care".[2-4] We used the Health Service Area because MA plan offerings, MA payment calculations, and provider networks all vary at the level of county (or groups of counties) and thus regression error terms are likely to be correlated.

References

1. Kim D, Makineni R, Panagiotou OA, et al. Assessment of Completeness of Hospital Readmission Rates Reported in Medicare Advantage Contracts' Healthcare Effectiveness Data and Information Set. *JAMA Network Open* 2020;3:e203555.

2. National Cancer Institute. Health Service Areas. https://seer.cancer.gov/seerstat/variables/countyattribs/hsa.html. Published 2008. Accessed.

3. Abadie A, Athey S, Imbens GW, Wooldridge JM. When Should You Adjust Standard Errors for Clustering? . *NBER Working Paper* 2017;24003.

4. Cameron AC, Miller DL. A Practioner's Guide to Cluster-Robust Inference. *The Journal of Human Resources.* 2015;50(2):317-372.

© 2024 Huckfeldt PJ et al. *JAMA Health Forum*

eResults

eFigure 1. Medicare Advantage enrollment for treatment and comparison group

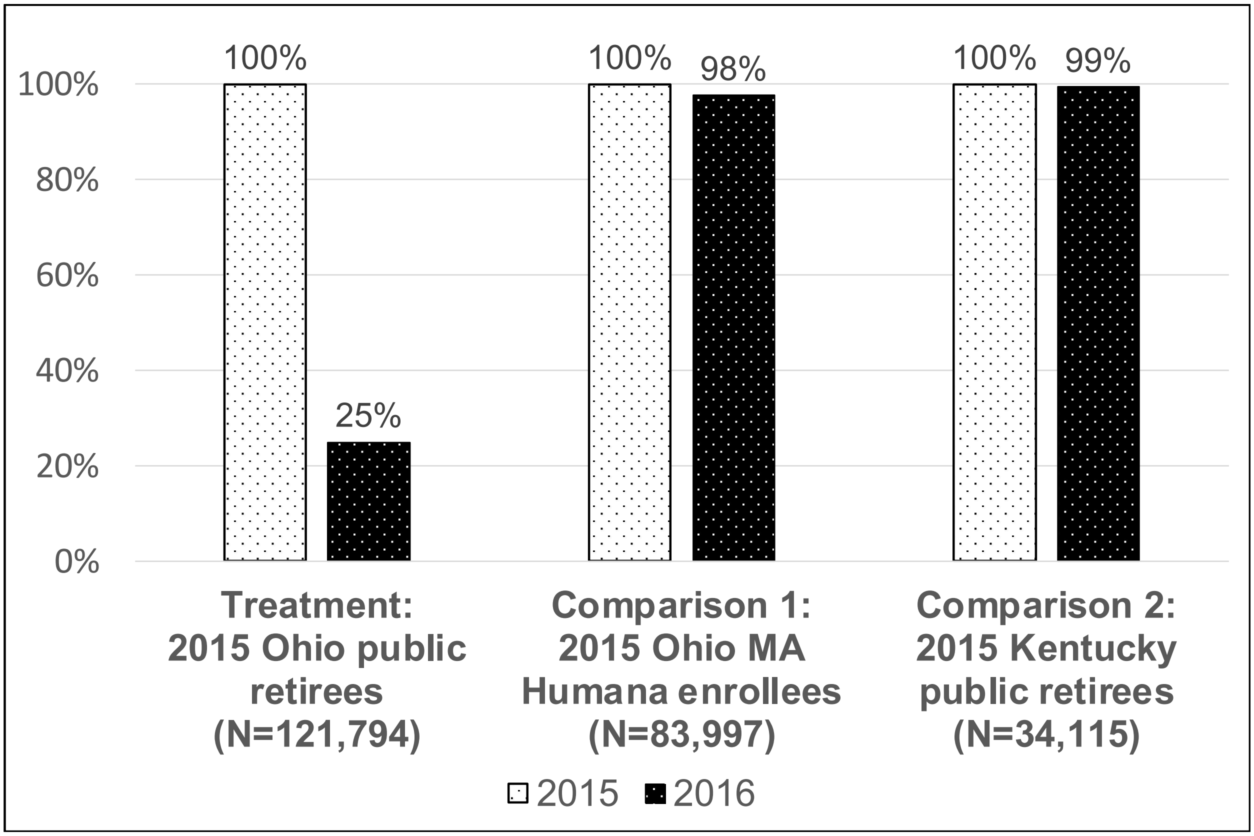

eTable 1. Difference in-difference regressions: Average change in quarterly hospitalizations per 1000 enrollees for Ohio public retirees relative to Comparison Groups

	Total hospitalizations (Across stroke and fractures)		Stroke hospitalizations		Hip and femur procedures (no joint replacement)		Fracture (with joint replacement)	
Quarterly hospitalizations per 1000 Ohio public retirees in 2015 (prior to switch to traditional Medicare)	2.62		1.34		0.84		0.42	
	Estimate (95% CI)	*P*	Estimate (95% CI)	*P*	Estimate (95% CI)	*P*	Estimate (95% CI)	*P*
Difference-in-difference estimate: Comparison group is other Ohio Humana MA enrollees **(Comparison group 1)**	0.03 (-0.23, 0.29)	0.801	0.09 (-0.07, 0.26)	0.251	-0.13 (-0.31, 0.06)	0.183	0.03 (-0.07, 0.14)	0.526
Difference-in-difference estimate: Comparison group is Kentucky public retirees **(Comparison group 2)**	-0.27 (-0.87, 0.33)	0.370	-0.08 (-0.28, 0.12)	0.437	-0.13 (-0.52, 0.27)	0.525	-0.04 (-0.21, 0.13)	0.641

Notes: Table displays average change in quarterly hospitalizations per 1000 in 2016 versus 2015 for Ohio public retirees relative to comparison groups.

eTable 2. Outcome variable means in 2015 and 2016

	Ohio public retirees (*Treatment Group)*		Other Humana MA enrollees in Ohio *(Comparison Group 1)*		2015 Kentucky public retirees (*Comparison Group 3)*	
	**2015 (n=1,217)**	**2016 (n=1,156)**	**2015 (n=840)**	**2016 (n=811)**	**2015 (n=285)**	**2016 (n=304)**
**Post-acute care use**						
Any IRF (%)	5.0	15.9	4.5	6.3	8.8	6.9
SNF or HH (no IRF) (%)	76.2	63.6	74.5	71.3	72.6	69.7
SNF with or without HH (%)	*65.6*	*54.7*	*60.7*	*57.5*	*56.8*	*54.6*
Only HH (%)	*10.6*	*8.9*	*13.8*	*13.8*	*15.8*	*15.1*
No PAC (%)	18.8	20.5	21.0	22.4	18.6	23.6
**Patient outcomes**						
30-day hospital readmission (%)	12.1	12.2	12.3	11.5	11.9	8.9
30-day hospital readmission (%) (Broader, using HEDIS)	13.9	13.6	13.2	12.8	13.0	11.1
Death during 30-day post discharge episode (%)	7.3	9.4	7.6	7.0	4.6	6.6
**Patient location in 30 days following hospital discharge**						
Days in the community	12.8	12.4	14.4	15.4	14.5	15.8
Days in institutional post-acute care	15.0	14.7	13.2	12.5	14.1	12.1
Days in hospital	0.7	0.8	0.7	0.7	0.5	0.7
Days deceased	1.5	2.0	1.6	1.4	0.9	1.5

Notes: Table displays the number of hospitalizations and outcome means for hospitalized patients in 2015 and 2016 separately for 2015 Ohio public retirees, the primary comparison group (Other Ohio Medicare beneficiaries enrolled in Humana MA plans in January 2015), and the secondary comparison group (2015 Kentucky public retirees).

© 2024 Huckfeldt PJ et al. *JAMA Health Forum*

eFigure 2. Event study estimate results: Medicare Advantage coverage, post-acute care use, and patient outcomes

a. Medicare Advantage coverage

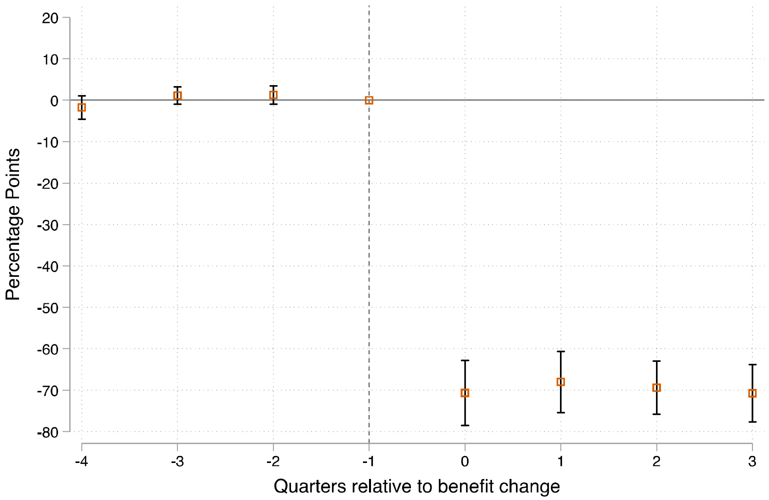

b. Any inpatient rehabilitation facility use

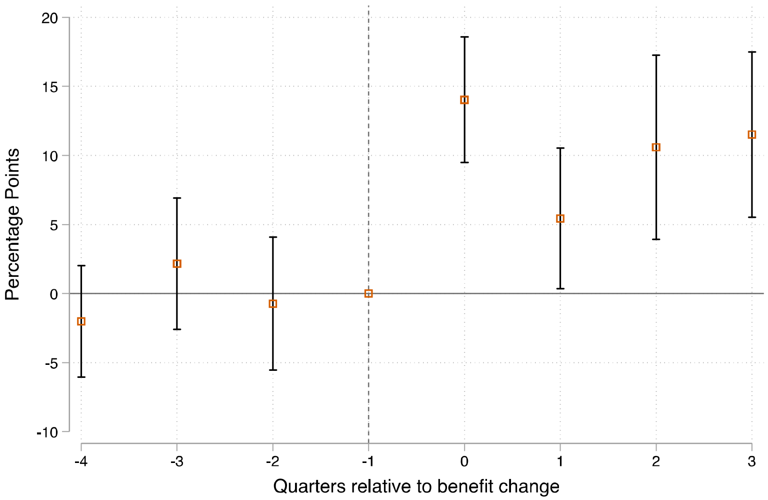

c. Skilled nursing facility (SNF) or home health (HHA), no IRF (%)

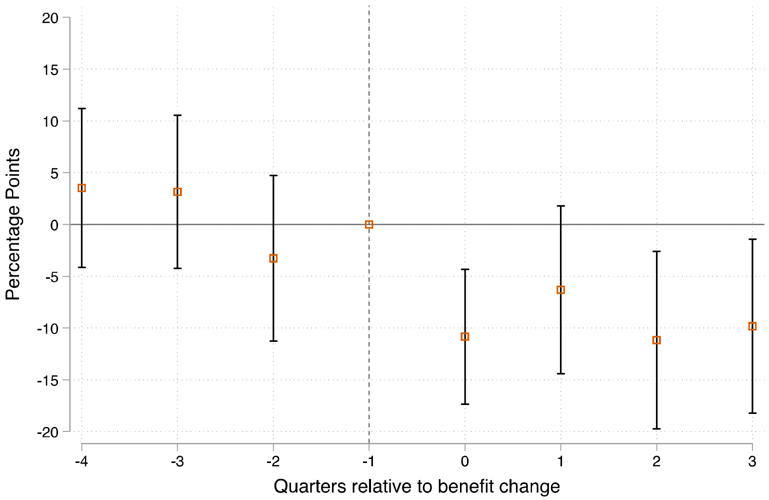

d. 30-day hospital readmissions

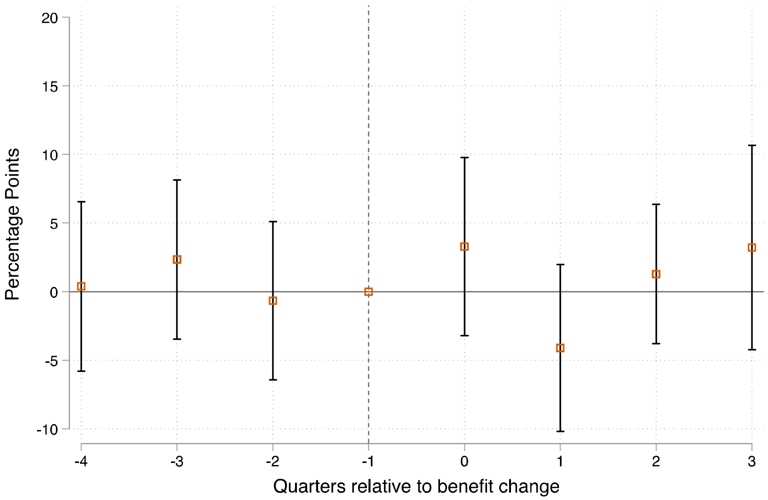

© 2024 Huckfeldt PJ et al. *JAMA Health Forum*

e. Days in the community (during 30-day post-discharge period)

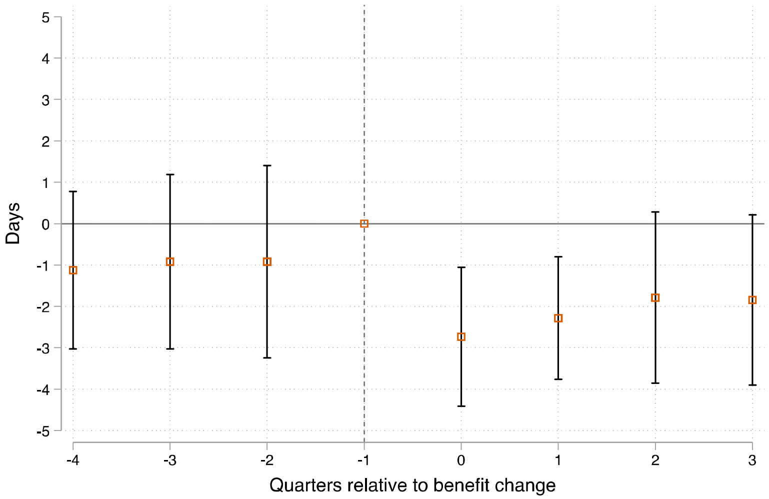

Notes: Event study coefficients (red squares) indicate the change in outcome for Ohio public retirees in each quarter relative to a comparison group of Ohio Medicare beneficiaries enrolled in other Humana plans in January 2015. The black brackets are 95% confidence interval. The reference period is the quarter prior the benefit change (2015 quarter 4).

© 2024 Huckfeldt PJ et al. *JAMA Health Forum*

eTable 3. Comparison of switchers and stayers, characteristics

	2016 Ohio public retirees (*Treatment group)*	
	**Switched to TM (n=862)**	**Stayed in MA (n=294)**
Age in years (SD)	81.3 (8.2)	81.6 (7.9)
Female N (%)	569 (66.0%)	199 (67.7%)
Race and ethnicity		
White, non-Hispanic N (%)	774 (89.8%)	261 (88.8%)
Index hospitalization		
Stroke N (%)	460 (53.4%)	150 (51.0%)
Fracture (without joint replacement) N (%)	263 (30.5%)	88 (29.9%)
Joint replacement due to fracture N (%)	139 (16.1%)	56 (19.1%)

© 2024 Huckfeldt PJ et al. *JAMA Health Forum*

eTable 4. Difference-in-difference estimates: Stratified by whether Ohio public retirees switched to traditional Medicare versus stayed in Medicare Advantage in 2016

a. Switched to traditional Medicare in 2016

		Difference-in-difference estimates: Ohio public retirees relative to comparison groups			
		Other Ohio Humana MA (Comparison Group 1)		Kentucky public retirees (Comparison Group 2)	
	Overall 2015 mean for Ohio public retirees	Estimate (95% CI)	*P* value	Estimate (95% CI)	*P* value
**Post-acute care use**					
Any IRF (%)	5.0	12.7 (6.9, 18.5)	0.000	15.0 (6.7, 23.4)	0.001
SNF or HH (no IRF) (%)	76.2	-12.2 (-19.1, -5.4)	0.001	-10.9 (-22.0, 0.2)	0.053
SNF with or without HH (%)	*65.6*	*-8.2 (-14.7, 1.6)*	*0.016*	*-6.5 (-14.8, 1.8)*	*0.122*
Only HH (%)	*10.6*	*-4.1 (-8.5, 0.4)*	*0.074*	*-4.4 (-12.3, 3.5)*	*0.268*
No PAC (%)	18.8	-0.5 (-5.1, 4.1)	0.831	-4.1 (-10.8, 2.5)	0.219
**Patient outcomes**					
30-day hospital readmissions (%)	12.1	-0.1 (-4.3, 4.2)	0.966	1.1 (-3.9, 6.2)	0.652
Death during 30-day post discharge episode (%)	7.3	0.7 (-2.0, 3.4)	0.587	-1.9 (-6.2, 2.3)	0.365
**Patient location in 30 days following hospital discharge**					
Days in the community	12.8	-2.1 (-3.4, -0.7)	0.003	-2.9 (-5.3, -0.5)	0.018
Days in institutional post-acute care	15.0	1.7 (0.4, 3.0)	0.014	3.6 (1.4, 5.8)	0.002
Days in the hospital	0.7	-0.04 (-0.3, 0.2)	0.740	-0.3 (-0.6, 0.1)	0.183
Days deceased	1.5	0.4 (-0.2, 1.0)	0.149	-0.4 (-1.4, 0.5)	0.398

Notes: The first column of the table displays the 2015 outcome mean for Ohio public retirees. The second panel displays the differences-in-differences estimates (with 95% confidence interval and p-values) showing relative changes in outcomes for Ohio public retirees versus Ohio Medicare beneficiaries enrolled in other Humana MA plans. The third panel displays the differences-in-differences estimates (with 95% confidence interval and p-values) showing relative changes in outcomes for Ohio public retirees versus Kentucky public retirees. Sample size was 3,730 hospital discharges for regressions comparing Ohio public retirees with other Ohio Humana MA enrollees and 2,668 hospital discharges for regressions comparing Ohio public retirees with Kentucky public retirees.

© 2024 Huckfeldt PJ et al. *JAMA Health Forum*

b. Stayed in Medicare Advantage in 2016

	Overall 2015 mean for Ohio public retirees	Difference-in-difference estimates: Ohio public retirees relative to comparison groups — Other Ohio Humana MA (Comparison Group 1): Estimate (95% CI)	*P* value	Kentucky public retirees (Comparison Group 2): Estimate (95% CI)	*P* value
**Post-acute care use**					
Any IRF (%)	5.0	0.7 (-2.9, 4.3)	0.699	2.7 (-4.0, 9.4)	0.419
SNF or HH (no IRF) (%)	76.2	2.1 (-3.0, 7.2)	0.415	3.5 (-6.5, 13.4)	0.487
SNF with or without HH (%)	*65.6*	*1.7 (-4.9, 8.3)*	*0.606*	*4.0 (-4.6, 12.7)*	*0.351*
Only HH (%)	*10.6*	*0.4 (-4.7, 5.5)*	*0.885*	*-0.6 (-8.6, 7.5)*	*0.887*
No PAC (%)	18.8	-2.8 (-7.4, 1.9)	0.236	-6.2 (-13.1, 0.8)	0.081
**Patient outcomes**					
30-day hospital readmissions (%)	12.1	3.6 (-1.6, 8.8)	0.166	5.4 (-0.6, 11.3)	0.076
Death during 30-day post discharge episode (%)	7.3	2.7 (-1.8, 7.2)	0.235	0.6 (-5.2, 6.4)	0.847
**Patient location in 30 days following hospital discharge**					
Days in the community	12.8	-0.4 (-2.0, 1.3)	0.659	-1.5 (-4.1, 1.1)	0.244
Days in institutional post-acute care	15.0	-0.4 (-1.9, 1.2)	0.644	1.8 (-0.7, 4.2)	0.153
Days in the hospital	0.7	0.3 (-0.03, 0.6)	0.079	0.03 (-0.4, 0.5)	0.895
Days deceased	1.5	0.5 (-0.5, 1.4)	0.314	-0.3 (-1.5, 1.0)	0.649

Notes: The first column of the table displays the 2015 outcome mean for Ohio public retirees. The second panel displays the differences-in-differences estimates (with 95% confidence interval and p-values) showing relative changes in outcomes for Ohio public retirees versus Ohio Medicare beneficiaries enrolled in other Humana MA plans. The third panel displays the differences-in-differences estimates (with 95% confidence interval and p-values) showing relative changes in outcomes for Ohio public retirees versus Kentucky public retirees. Sample size was 3,162 hospital discharges for regressions comparing Ohio public retirees with other Ohio Humana MA enrollees and 2,100 hospital discharges for regressions comparing Ohio public retirees with Kentucky public retirees.

© 2024 Huckfeldt PJ et al. *JAMA Health Forum*

eTable 5. Difference-in-difference estimates: Stratified by condition

a. Stroke

		Difference-in-difference estimates: Ohio public retirees relative to comparison groups			
		Other Ohio Humana MA (Comparison Group 1)		Kentucky public retirees (Comparison Group 2)	
	Overall 2015 mean for Ohio public retirees	Estimate (95% CI)	*P* value	Estimate (95% CI)	*P* value
Medicare Advantage coverage (%)	>99%	-69.9 (-75.8, -64.1)	0.000	-72.9 (-79.6, -66.3)	0.000
**Post-acute care use**					
Any IRF (%)	5.0	12.2 (5.6, 18.9)	0.001	13.0 (-0.1, 26.1)	0.051
SNF or HH (no IRF)	76.2	-5.5 (-14.0, 3.1)	0.204	-5.3 (-20.0, 9.4)	0.476
SNF with or without HH (%)	*65.6*	*-2.7 (-11.1, 5.8)*	*0.527*	*-3.7 (15.1, 7.7)*	*0.518*
Only HH (%)	*10.6*	*-2.8 (-10.1, 4.5)*	*0.449*	*-1.6 (-12.4, 9.3)*	*0.772*
No PAC (%)	18.8	-6.7 (-13.6, 0.2)	0.055	-7.7 (-18.2, 2.7)	0.144
**Patient outcomes**					
30-day hospital readmissions (%)	12.1	3.8 (-3.0, 10.7)	0.262	-0.2 (-7.8, 7.4)	0.959
Death during 30-day post discharge episode (%)	7.3	1.7 (-2.8, 6.2)	0.446	-2.4 (-8.2, 3.3)	0.397
**Patient location in 30 days following hospital discharge**					
Days in the community	12.8	-2.4 (-4.2, -0.7)	0.008	-2.0 (-5.5, 1.5)	0.264
Days in institutional post-acute care	15.0	1.8 (0.2, 3.5)	0.030	3.1 (-0.3, 6.5)	0.072
Days in the hospital	0.7	0.2 (-0.2, 0.6)	0.358	-0.4 (-0.9, 0.2)	0.168
Days deceased	1.5	0.4 (-0.6, 1.4)	0.393	-0.7 (-2.2, 0.7)	0.302

Notes: The first column of the table displays the 2015 outcome mean for Ohio public retirees. The second panel displays the differences-in-differences estimates (with 95% confidence interval and p-values) showing relative changes in outcomes for Ohio public retirees versus Ohio Medicare beneficiaries enrolled in other Humana MA plans. The third panel displays the differences-in-differences estimates (with 95% confidence interval and p-values) showing relative changes in outcomes for Ohio public retirees versus Kentucky public retirees. Sample size was 2,105 hospital discharges for regressions comparing Ohio public retirees with other Ohio Humana MA enrollees and 1.505 hospital discharges for regressions comparing Ohio public retirees with Kentucky public retirees.

© 2024 Huckfeldt PJ et al. *JAMA Health Forum*

© 2024 Huckfeldt PJ et al. *JAMA Health Forum*

b. Fracture (with or without joint replacement)

	Overall 2015 mean for Ohio public retirees	Difference-in-difference estimates: Ohio public retirees relative to comparison groups — Other Ohio Humana MA (Comparison Group 1): Estimate (95% CI)	*P* value	Kentucky public retirees (Comparison Group 2): Estimate (95% CI)	*P* value
Medicare Advantage coverage (%)	>99%	-69.6 (-74.1, -65.1)	0.000	-73.6 (-78.5, -68.6)	0.000
**Post-acute care use**					
Any IRF (%)	5.0	7.0 (2.0, 12.0)	0.007	10.8 (4.5, 17.0)	0.001
SNF or HH (no IRF)	76.2	-10.8 (-17.0, -4.6)	0.001	-10.6 (-18.8, -2.4)	0.012
SNF with or without HH (%)	*65.6*	*-8.6 (-16.2, -1.1)*	*0.025*	*-4.1 (-14.6, 6.5)*	*0.443*
Only HH (%)	*10.6*	*-2.2 (-7.0, 2.7)*	*0.376*	*-6.5 (-16.4, 3.4)*	*0.191*
No PAC (%)	18.8	3.8 (-0.8, 8.4)	0.105	-0.2 (-5.8, 5.5)	0.953
**Patient outcomes**					
30-day hospital readmissions (%)	12.1	-1.6 (-6.5, 3.3)	0.517	4.4 (-3.0, 11.8)	0.239
Death during 30-day post discharge episode (%)	7.3	-0.2 (-4.5, 4.1)	0.917	-0.1 (-4.3, 4.1)	0.966
**Patient location in 30 days following hospital discharge**					
Days in the community	12.8	-0.6 (-2.4, 1.1)	0.473	-3.1 (-6.0, -0.3)	0.030
Days in institutional post-acute care	15.0	0.5 (-1.3, 2.3)	0.572	3.0 (0.6, 5.4)	0.016
Days in the hospital	0.7	-0.1 (-0.6, 0.4)	0.682	-0.02 (-0.8, 0.8)	0.955
Days deceased	1.5	0.2 (-0.6, 1.0)	0.584	0.2 (-0.7, 1.1)	0.730

Notes: The first column of the table displays the 2015 outcome mean for Ohio public retirees. The second panel displays the differences-in-differences estimates (with 95% confidence interval and p-values) showing relative changes in outcomes for Ohio public retirees versus Ohio Medicare beneficiaries enrolled in other Humana MA plans. The third panel displays the differences-in-differences estimates (with 95% confidence interval and p-values) showing relative changes in outcomes for Ohio public retirees versus Kentucky public retirees. Sample size was 1,919 hospital discharges for regressions comparing Ohio public retirees with other Ohio Humana MA enrollees and 1,457 hospital discharges for regressions comparing Ohio public retirees with Kentucky public retirees.

© 2024 Huckfeldt PJ et al. *JAMA Health Forum*

eTable 6. Difference-in-difference estimates: Stratified by metropolitan versus non-metropolitan residence

a. Resides in metropolitan county

		Difference-in-difference estimates: Ohio public retirees relative to comparison groups			
		Other Ohio Humana MA (Comparison Group 1)		Kentucky public retirees (Comparison Group 2)	
	Overall 2015 mean for Ohio public retirees	Estimate (95% CI)	*P* value	Estimate (95% CI)	*P* value
Medicare Advantage coverage (%)	>99%	-69.5 (-74.4, -64.7)	0.000	-72.8 (-78.7, -66.9)	0.000
**Post-acute care use**					
Any IRF (%)	5.0	10.5 (5.0, 16.1)	0.000	11.7 (2.7, 20.7)	0.013
SNF or HH (no IRF)	76.2	-7.5 (-14.1, -0.9)	0.027	-6.5 (-20.2, 7.3)	0.345
SNF with or without HH (%)	*65.6*	*-4.3 (-10.7, 2.1)*	*0.181*	*-1.0 (-11.0, 9.0)*	*0.843*
Only HH (%)	*10.6*	*-3.2 (-8.4, 2.0)*	*0.218*	*-5.5 (-18.0, 7.0)*	*0.377*
No PAC (%)	18.8	-3.0 (-8.1, 2.0)	0.231	-5.2 (-14.0, 3.5)	0.233
**Patient outcomes**					
30-day hospital readmissions (%)	12.1	1.3 (-3.2, 5.8)	0.553	2.7 (-1.9, 7.3)	0.242
Death during 30-day post discharge episode (%)	7.3	0.8 (-2.4, 4.1)	0.616	1.5 (-7.3, 4.2)	0.586
**Patient location in 30 days following hospital discharge**					
Days in the community	12.8	-2.0 (-3.5, -0.5)	0.010	-2.6 (-5.6, 0.3)	0.076
Days in institutional post-acute care	15.0	1.6 (0.3, 2.9)	0.014	2.9 (-0.3, 6.1)	0.071
Days in the hospital	0.7	0.05 (-0.2, 0.3)	0.664	-0.01 (-0.4, 0.4)	0.941
Days deceased	1.5	0.3 (-0.3, 1.0)	0.312	-0.3 (-1.6, 1.1)	0.676

Notes: The first column of the table displays the 2015 outcome mean for Ohio public retirees. The second panel displays the differences-in-differences estimates (with 95% confidence interval and p-values) showing relative changes in outcomes for Ohio public retirees versus Ohio Medicare beneficiaries enrolled in other Humana MA plans. The third panel displays the differences-in-differences estimates (with 95% confidence interval and p-values) showing relative changes in outcomes for Ohio public retirees versus Kentucky public retirees. Sample size was 3,026 hospital discharges for regressions comparing Ohio public retirees with other Ohio Humana MA enrollees and 2,116 hospital discharges for regressions comparing Ohio public retirees with Kentucky public retirees.

© 2024 Huckfeldt PJ et al. *JAMA Health Forum*

b. Resides in non-metropolitan county

		Difference-in-difference estimates: Ohio public retirees relative to comparison groups			
		Other Ohio Humana MA (Comparison Group 1)		Kentucky public retirees (Comparison Group 2)	
	Overall 2015 mean for Ohio public retirees	Estimate (95% CI)	*P* value	Estimate (95% CI)	*P* value
Medicare Advantage coverage (%)	>99%	-69.2 (-76.9, -61.4)	0.000	-74.8 (-81.8, -67.7)	0.000
**Post-acute care use**					
Any IRF (%)	5.0	8.3 (-0.3, 16.9)	0.058	15.2 (2.5, 28.0)	0.020
SNF or HH (no IRF) (%)	76.2	-15.1 (-26.4, -3.8)	0.010	-12.4 (-29.7, 4.9)	0.156
SNF with or without HH (%)	*65.6*	*-14.6 (-28.1, -1.1)*	*0.035*	*-11.2 (-29.6, 7.2)*	*0.226*
Only HH (%)	*10.6*	*-0.5 (-9.1, 8.1)*	*0.906*	*-1.2 (-13.8, 11.4)*	*0.850*
No PAC (%)	18.8	6.8 (-0.8, 14.4)	0.077	-2.8 (-16.9, 11.2)	0.686
**Patient outcomes**					
30-day hospital readmissions (%)	12.1	0.3 (-7.7, 8.2)	0.949	-0.03 (-7.4, 7.4)	0.993
Death during 30-day post discharge episode (%)	7.3	4.2 (-1.9, 10.3)	0.170	-1.6 (-8.8, 5.6)	0.659
**Patient location in 30 days following hospital discharge**					
Days in the community	12.8	0.3 (-2.4, 3.0)	0.812	-2.2 (-7.2, 2.8)	0.376
Days in institutional post-acute care	15.0	-1.7 (-4.8, 1.5)	0.298	3.0 (-1.8, 7.9)	0.215
Days in the hospital	0.7	0.2 (-0.6, 1.0)	0.667	-0.4 (-1.2, 0.5)	0.384
Days deceased	1.5	1.2 (-0.2, 2.6)	0.100	-0.4 (-2.0, 1.2)	0.585

Notes: The first column of the table displays the 2015 outcome mean for Ohio public retirees. The second panel displays the differences-in-differences estimates (with 95% confidence interval and p-values) showing relative changes in outcomes for Ohio public retirees versus Ohio Medicare beneficiaries enrolled in other Humana MA plans. The third panel displays the differences-in-differences estimates (with 95% confidence interval and p-values) showing relative changes in outcomes for Ohio public retirees versus Kentucky public retirees. Sample size was 998 hospital discharges for regressions comparing Ohio public retirees with other Ohio Humana MA enrollees and 846 hospital discharges for regressions comparing Ohio public retirees with Kentucky public retirees.

© 2024 Huckfeldt PJ et al. *JAMA Health Forum*
