# Supplementary material for: Postacute Care for Medicare Advantage Enrollees Who Switched to Traditional Medicare Compared With Those Who Remained in Medicare Advantage
Source: JAMA Health Forum. 2024 Feb 16;5(2):e235325. doi: 10.1001/jamahealthforum.2023.5325 (PMC10873769; doi:10.1001/jamahealthforum.2023.5325)
Supplement: Supplement 2. — Data Sharing Statement [file jamahealthforum-e235325-s002.pdf]

Data Sharing Statement

Huckfeldt. Postacute Care for Medicare Advantage Enrollees Who Switched to Traditional Medicare Compared With Those Who Remained in Medicare Advantage. *JAMA Health Forum*. Published February 16, 2024. doi:10.1001/jamahealthforum.2023.5325

Data
Data available: No

Additional Information
Explanation for why data not available: We have a data use agreement with the Centers for Medicare and Medicaid Services that prohibits data sharing.
